# Neuroinvasive West Nile Virus Infection in Immunosuppressed and Immunocompetent Adults

**DOI:** 10.1001/jamanetworkopen.2024.4294

**Published:** 2024-03-28

**Authors:** Amir A. Mbonde, David Gritsch, Ehab Y. Harahsheh, Sabirah N. Kasule, Shemonti Hasan, Angela M. Parsons, Nan Zhang, Richard Butterfield, Harn Shiue, Kathryn A. Norville, Jenna L. Reynolds, Holenarasipur R. Vikram, Brian Chong, Marie F. Grill

**Affiliations:** 1 Department of Neurology, Mayo Clinic College of Medicine and Science, Phoenix, Arizona; 2 Department of Neurology, Massachusetts General Hospital, Boston; 3 Harvard Medical School, Boston, Massachusetts; 4 Division of Infectious Diseases, Mayo Clinic College of Medicine and Science, Phoenix, Arizona; 5 OhioHealth Physicians Group, Columbus; 6 Department of Quantitative Health Sciences, Division of Clinical Trials and Biostatistics, Mayo Clinic, Phoenix, Arizona; 7 Department of Pharmacy, Mayo Clinic College of Medicine and Science, Phoenix, Arizona; 8 Department of Neuroradiology, Mayo Clinic College of Medicine and Science, Phoenix, Arizona

## Abstract

**Importance:**

West Nile virus (WNV) is the leading cause of human arboviral disease in the US, peaking during summer. The incidence of WNV, including its neuroinvasive form (NWNV), is increasing, largely due to the expanding distribution of its vector, the *Culex* mosquito, and climatic changes causing heavy monsoon rains. However, the distinct characteristics and outcomes of NWNV in individuals who are immunosuppressed (IS) and individuals who are not IS remain underexplored.

**Objective:**

To describe and compare clinical and radiographic features, treatment responses, and outcomes of NWNV infection in individuals who are IS and those who are not IS.

**Design, Setting, and Participants:**

This retrospective cohort study used data from the Mayo Clinic Hospital system collected from July 2006 to December 2021. Participants were adult patients (age ≥18 years) with established diagnosis of NWNV infection. Data were analyzed from May 12, 2020, to July 20, 2023.

**Exposure:**

Immunosuppresion.

**Main Outcomes and Measures:**

Outcomes of interest were clinical and radiographic features and 90-day mortality among patients with and without IS.

**Results:**

Of 115 participants with NWNV infection (mean [SD] age, 64 [16] years; 75 [66%] male) enrolled, 72 (63%) were not IS and 43 (37%) were IS. Neurologic manifestations were meningoencephalitis (98 patients [85%]), encephalitis (10 patients [9%]), and myeloradiculitis (7 patients [6%]). Patients without IS, compared with those with IS, more frequently reported headache (45 patients [63%] vs 18 patients [42%]) and myalgias (32 patients [44%] vs 9 patients [21%]). In contrast, patients with IS, compared with those without, had higher rates of altered mental status (33 patients [77%] vs 41 patients [57%]) and myoclonus (8 patients [19%] vs 8 patients [4%]). Magnetic resonance imaging revealed more frequent thalamic T2 fluid-attenuated inversion recovery hyperintensities in individuals with IS than those without (4 patients [11%] vs 0 patients). Individuals with IS had more severe disease requiring higher rates of intensive care unit admission (26 patients [61%] vs 24 patients [33%]) and mechanical ventilation (24 patients [56%] vs 22 patients [31%]). The 90-day all-cause mortality rate was higher in the patients with IS compared with patients without IS (12 patients [28%] vs 5 patients [7%]), and this difference in mortality persisted after adjusting for Glasgow Coma Scale score (adjusted hazard ratio, 2.22; 95% CI, 1.07-4.27; *P* = .03). Individuals with IS were more likely to receive intravenous immunoglobulin than individuals without IS (12 individuals [17%] vs 24 individuals [56%]), but its use was not associated with survival (hazard ratio, 1.24; 95% CI, 0.50-3.09; *P* = .64).

**Conclusions and Relevance:**

In this cohort study of individuals with NWNV infection, individuals with IS had a higher risk of disease complications and poor outcomes than individuals without IS, highlighting the need for innovative and effective therapies to improve outcomes in this high-risk population.

## Key Points

**Question:**

Do clinical manifestations, radiographic characteristics, and outcomes of neuroinvasive West Nile virus (NWNV) infection differ between adult patients with vs without immunosuppression (IS)?

**Findings:**

In this cohort study of 115 patients with NWNV infection, there were significant differences in clinical presentations between patients with vs without IS. Patients with IS experienced more severe disease, indicated by higher rates of intensive care unit admissions, mechanical ventilation, and 90-day all-cause mortality, compared with patients without IS.

**Meaning:**

These findings suggest that individuals with IS with NWNV presented with more severe clinical manifestations and had poorer outcomes compared with individuals without IS, highlighting the need for targeted disease prevention and treatment strategies for this high-risk group.

## Teaser

This cohort study describes and compares clinical and radiographic features, treatment responses, and outcomes of neuroinvasive West Nile virus infection in individuals with and without immunosuppression.

## Introduction

West Nile virus (WNV), an arbovirus of the *Flaviviridae* family, has rapidly spread across the United States since its first appearance in 1999 in New York, New York. By 2007, the disease had been reported in 44 states within the US and subsequently in all 50 states.^1,2,3^ In 2021, several states on the western coast of the US experienced an unprecedented outbreak of WNV infections, most notably Arizona, which reported an incredibly high number of cases, with more than 1700 individuals infected, including 575 neuroinvasive WNV (NWNV) infections and 125 deaths.^4^ This surge has been attributed to an increase in the distribution of its vector, the *Culex* mosquito (with birds serving as the main viral amplifying host) and climate changes leading to heavy monsoon rains.^5^

While most WNV infections are asymptomatic, less than 1% of individuals develop NWNV infection, a potentially lethal condition encompassing encephalitis, meningitis, myelitis, and radiculitis.^6^ Between 1999 and 2019, there were 25 920 cases of NWNV infection reported in the United States, of which 2259 were fatal.^1,6^ Limited reports have suggested that individuals who are immunosuppressed (IS), such as recipients of solid organ transplant (SOT), may exhibit atypical clinical features of NWNV infection, leading to delayed diagnosis and potentially worse outcomes.^7,8,9^

Despite the increasing prevalence of IS-related conditions in the US,^10^ there is a significant gap in the literature detailing the clinical presentation, radiographic manifestations, and outcomes of NWNV infection in patients with IS vs those who are not IS. Identifying any discerning clinical features could enhance our understanding of NWNV disease, improve early recognition, and potentially lead to timely interventions in this high-risk group of individuals. In this study, we aimed to describe the presenting clinical features, radiographic characteristics, treatment interventions, and outcomes in individuals with and without IS with NWNV infection seen at the Mayo Clinic hospitals over a 10-year period, including patients from the 2021 Maricopa County, Arizona, outbreak.

## Methods

### Ethical Considerations

This cohort study was approved by the Mayo Clinic institutional review board. Informed consent was not obtained from individual participants, as the institutional review board granted an exemption for this requirement because only deidentified data were extracted from the patient electronic medical records. This study is reported following the Strengthening the Reporting of Observational Studies in Epidemiology (STROBE) reporting guideline.

### Search Criteria and Case Selection

We executed a comprehensive search of the Mayo Clinic Enterprise electronic medical record platform spanning the period July 2006 to September 2019. Our search was broad, including all patients with WNV infection treated at any of the Mayo clinic hospitals in Phoenix, Arizona; Jacksonville, Florida; and Rochester, Minnesota. This search used *International Classification of Diseases, Ninth Revision *(*ICD-9*) codes (066.40, 066.41, 066.42, 066.49) and *International Statistical Classification of Diseases and Related Health Problems, Tenth Revision *(*ICD-10*) codes (A92.30, A92.31, A92.32) for WNV infection. All records identified following this initial search were reviewed for inclusion. We included all adult individuals (age ≥18 years) with a diagnosis of NWNV infection, defined as patients with documented diagnosis of isolated encephalitis, meningitis with or without encephalitis (meningoencephalitis), or acute flaccid myelitis with or without radiculitis (myeloradiculitis) in the setting of laboratory-established WNV infection (positive serum or cerebrospinal fluid [CSF] WNV immunoglobin M [IgM] and/or positive CSF WNV RNA polymerase chain reaction [PCR] test result). Following the 2021 WNV outbreak in Arizona, we conducted a second search to include only individuals from Arizona for the year 2021. The entire dataset includes patients enrolled in a previously published study of 24 recipients of SOT with NWNV infection.^9^

Reasons for exclusion of participants were history of WNV infection or prior documented diagnosis and treatment for WNV unrelated to admission at Mayo Clinic (outside hospital records not available), clinical suspicion for WNV infection but absent supportive diagnostic serum and CSF studies, acute WNV infection and positive serology test result without CNS involvement; and patient declined to offer research authorization at time of hospitalization, which is a requirement for Minnesota residents (eFigure 1 in Supplement 1). Individuals with NWNV infection who were receiving immunosuppressive medications, including high-dose prednisone, calcineurin inhibitors, biologics (eg, immune check point inhibitors, tumor necrosis factor–α inhibitors, Bruton tyrosine kinase inhibitors, and rituximab), or any cytotoxic chemotherapy agent, were considered IS. In addition, individuals with a history of SOT, stem cell transplant, and active cancer were considered IS. Individuals without any of these comorbid conditions were classified as not IS.

Magnetic resonance imaging (MRI) results were considered negative if there were no acute findings. Significant acute abnormal MRI findings were defined as T2 fluid-attenuated inversion recovery (FLAIR) abnormalities, acute infarct (as evidenced by high diffusion-weighted imaging signal and low apparent diffusion coefficient), acute intracranial hemorrhage, and leptomeningeal enhancement.

### Data Collection

Relevant data were obtained using a data extraction form, including sociodemographic characteristics, clinical signs and symptoms, laboratory test results, radiographic findings, treatments received, and outcome data, including hospital length of stay and survival status at 90 days, as recorded in the medical record. There were no losses to follow up. Data were extracted by 3 residents and 2 fellows in the departments of neurology and infectious diseases (A.A.M., D.G., E.Y.H., S.N.K., S.H., and A.M.P.) and hospital pharmacy staff (H.S., K.A.N., and J.L.R.). All extracted data were directly entered into a REDCap (Vanderbilt University) database questionnaire hosted on the Mayo Clinic server. Data from MRI images were directly extracted from the corresponding radiology reports in the patient files, and all abnormal MRI findings were independently verified by a board certified neuroradiologist (B.C.).

### Statistical Analysis

Statistical analyses were performed by 2 independent experts from the Mayo Clinic bioinformatics core (N.Z. and R.B.). Missing data were treated as missing and reported where applicable. Logistic regression analysis was performed to identify associations with 90-day mortality. Due to a sparse number of mortality events, only 3 variables were added to the final model. Variables with *P* ≤ 0.05 were included in the final regression model. Variables that were clinically associated with low Glasgow Coma Scale (GCS), such as intensive care unit admission, mechanical ventilation, and tracheostomy, were excluded from the multivariable model, and instead, low GCS was selected due to its clinically relevant association with disease severity. Intravenous immunoglobulin (IVIg) use and IS status were also associated with each other; thus IVIg use was not included as a variable in the model. Additional analysis was completed in a subgroup of individuals with IS to determine whether IVIg use was associated with reduced risk of mortality. P values were 2-sided, and statistical significance was set at *P* < .05. Analyses were conducted using SAS version 9.4 (SAS Institute). Data were analyzed from May 12, 2020, to July 20, 2023.

## Results

### Sociodemographic Characteristics

Of 115 patients with NWNV infection (mean [SD] age, 64 [16] years; 75 [66%] male) included, we classified 72 (63%) as not IS and 43 (37%) as IS. Baseline sociodemographic characteristics did not differ significantly between individuals with vs without IS (Table 1). However, individuals with IS had a significantly delayed hospital presentation, with median (IQR) presentation time from symptom onset of 7 (4-14) days in patients with IS vs 5 (3-7) days in patients without IS (Table 1). Overall, most patients presented in the summer (56 patients [49%]) and fall (56 patients [49%]) months (Table 1; eFigure 2 in Supplement 1). There were 38 patients (33%) in Minnesota, 2 patients (2%) in Florida and 75 patients (65%) in Arizona (Table 1).

**Table 1.** Sociodemographic and Clinical Characteristics

Characteristics	Patients, No. (%)		
	Not immunosuppressed (n = 72)	Immunosuppressed (n = 43)	Total (N = 115)
Age, mean (SD), y	63 (17)	65 (14)	64 (16)
Sex			
Male	49 (68)	26 (60)	75 (66)
Female	23 (32)	17 (40)	39 (34)
Season of presentation			
Summer	36 (50)	20 (47)	56 (49)
Fall	34 (47)	22 (51)	56 (49)
Duration of symptoms, median (IQR), d [No. missing]	5 (3-7) [1]	7 (4-14) [3]	6 (3-9) [4]
Symptoms and signs			
Malaise	55 (76)	28 (65)	83 (72)
Fever	50 (70)	26 (61)	76 (66)
Headache	45 (63)	18 (42)	63 (55)
Altered mental status	41 (57)	33 (77)	74 (64)
Diarrhea or vomiting [No. missing]	33 (47) [1]	21 (49) [0]	54 (48) [1]
Myalgias	32 (44)	9 (21)	41 (36)
Hand tremors	24 (33)	13 (30)	37 (32)
Skin rash	17 (24)	10 (23)	27 (24)
Focal motor signs [No. missing]	12 (17) [1]	13 (30) [0]	25 (22) [1]
Cranial nerve signs	5 (7)	6 (14)	11 (10)
Myoclonus	3 (4)	8 (19)	11 (10)
Clinical seizure	4 (8)	6 (19)	10 (12)
Coma (GCS<8)	14 (19)	15 (35)	29 (25)
WNV-specific laboratory findings [No. missing]			
Serum WNV IgM, positive	61 (78) [5]	33 (81) [2]	94 (87) [7]
CSF WNV IgM, positive	47 (78) [12]	24 (62) [4]	71 (72) [16]
CSF WNV PCR, positive	5 (15) [39]	17 (63) [16]	22 (37) [55]
CSF WBC count, median (IQR), /μL	113 (62-355) [7]	79 (20-144) [5]	90 (41-229) [12]
CSF protein, median (IQR), mg/dL	86 (65-104) [8]	75 (52-99) [6]	82 (61-101) [14]
CSF glucose, median (IQR), mg/dL	59 (53-72) [7]	64 (57-72) [8]	61 (54-72) [15]
Final diagnosis			
WNV meningoencephalitis	65 (90)	33 (77)	98 (85)
WNV encephalomyelitis	3 (4)	7 (16)	10 (9)
WNV myeloradiculitis	4 (6)	3 (7)	7 (6)

Abbreviations: CSF, cerebrospinal fluid; GCS, Glasgow Coma Scale; IgM, immunoglobulin M; PCR, polymerase chain reaction; WBC, white blood cell; WNV, West Nile virus.

SI conversion factor: To convert WBC to ×10^9^/L, multiply by 0.001.

### Clinical Features, Disease Severity, and Diagnosis at Presentation

The most common clinical signs and symptoms in the total cohort were malaise (83 patients [72%]), fever (76 patients [66%]), headache (63 patients [55%]), altered mentation (74 patients [64%]), diarrhea and/or vomiting (54 patients [48%]), myalgias (41 patients [36%]), and focal motor signs, such as monoparesis, hemiparesis, or quadriparesis (25 patients [22%]). Headaches were more common in individuals without IS, occurring in 45 patients (63%), compared with 18 patients (42%) with IS. Similarly, myalgias were reported by 32 patients (44%) without IS, compared with 9 patients (21%) with IS. Conversely, altered mental status (33 patients [77%] vs 41 patients [57%]) and myoclonus (8 patients [19%] vs 3 patients [4%]) were more frequently observed in individuals with IS than patients without IS.

The most common diagnosis in the total cohort was WNV meningoencephalitis (98 patients [85%]), followed by WNV encephalitis (10 patients [9%]) and WNV myeloradiculitis in (7 patients [6%]). The frequency of these diagnoses did not differ between individuals with vs without IS. Patients with IS, compared with those without, were more frequently admitted to the intensive care unit (26 patients [61%] vs 24 patients [33%]) and more frequently received mechanical ventilation (24 patients [56%] vs 22 patients [31%]) (Table 2).

**Table 2.** Treatment and Outcomes of Individuals With and Without Immunosuppression With Neuroinvasive West Nile Virus Infection

Adjunctive therapy	Patients, No. (%)		
	Not immunosuppressed (n = 72)	Immunosuppressed (n = 43)	Total (N = 115)
None	52 (72)	15 (35)	67 (58)
Any single agent (IVIg, interferon-alfa, ribavirin)	16 (22)	13 (30)	29 (25)
Any combination of agents	4 (6)	15 (35)	19 (16)
Interferon-alfa	5 (7)	16 (37)	21 (18)
IVIg	12 (17)	24 (56)	36 (31)
Other managements			
ICU admission	24 (33)	26 (61)	50 (44)
Mechanical ventilation	22 (31)	24 (56)	46 (41)
Outcome			
Hospital length of stay, median (IQR), d [No. missing]	8 (5-20) [1]	16 (11-26) [0]	12 (6-23) [1]
90-Day mortality,	5 (7)	12 (28)	17 (15)

Abbreviations: ICU, intensive care unit; IVIg, intravenous immunoglobulin.

### Diagnostic Information

In the total cohort of 115 individuals, 94 (87%) had positive serum WNV IgM antibodies and 71 (72%) had positive CSF WNV IgM antibodies, with 61 of 92 participants who received both laboratory studies (66%) having concurrent positive CSF and positive serum WNV IgM antibodies. The serum and CSF WNV IgM antibody positivity rates were similar between groups. However, a positive CSF WNV PCR result was more common in individuals with IS than individuals without IS (17 patients [63%] vs 5 patients [15%]). CSF white blood cell count was significantly higher in individuals without IS compared with individuals with IS (median [IQR], 90 [41-229]/μL vs 79 [20-144]/μL; to convert to ×10^9^/L, multiply by 0.001).

MRI data were available in 78 individuals (43 individuals without IS; 35 individuals with IS), and MRI findings were normal in 29 individuals (37%) in the entire cohort. T2 FLAIR hyperintensities were detected in 37 patients (47%) in the total cohort, with the most common locations being the brainstem (28 patients [36%]), thalamus (4 patients [5%]) and mesial temporal lobes (3 patients [4%]) (Table 3 and Figure). Overall, the distribution of T2 FLAIR hyperintensities was comparable between groups, except for thalamic lesions, which were more common in patients with IS than those without IS (4 patients [11%] vs 0 patients). Punctate acute infarcts were noted in 11 patients (14%) in the total cohort, and the most common locations were the cerebral cortex (2 patients [3%]), basal ganglia (3 patients [4%]), and pons (3 patients [4%]) (Figure), but this did not differ between groups (Table 3).

**Table 3.** Magnetic Resonance Imaging Findings

Finding	Patients, No. (%)		
	Not immunosuppressed (n = 43)	Immunosuppressed (n = 35)	Total (N = 78)
No abnormal findings	17 (40)	12 (34)	29 (37)
Acute T2 FLAIR hyperintensities	18 (42)	19 (54)	37 (47)
Brain stem (cerebral peduncle, pons, midbrain)	13 (30)	15(43)	28 (36)
Thalamus	0	4 (11)	4 (5)
Mesial temporal lobe	2 (5)	1 (3)	3 (4)
Basal ganglia	0	1 (3)	1 (1)
Cortical regions	1 (3)	0	1 (1)
Cerebellum	1 (2)	1 (3)	2 (3)
Spinal cord	1 (2)	0	1 (1)
Leptomeningeal enhancement	7 (16)	6 (17)	13 (17)
Cauda equina and nerve roots	0	1 (3)	1 (1)
Cortical regions	3 (7)	3 (9)	6 (8)
Cerebellum	0	1 (3)	1 (1)
Diffuse dural enhancement	1 (2)	0	1 (1)
Acute infarct^a^	7 (16)	4 (11)	11 (14)
Cortical region infarct	2 (5)	0	2 (3)
Basal ganglia, lacuna infarct	2 (5)	1 (3)	3 (4)
Pontine infarct	2 (5)	1 (3)	3 (4)
Cerebellum	0	1 (3)	1 (1)
Acute diffusion restriction in centrum semiovale and corpus callosum	4 (9)	1 (3)	5 (6)

Abbreviation: FLAIR, fluid-attenuated inversion recovery.

^a^ All areas of acute ischemic or diffusion restriction were tiny.

**Figure.**
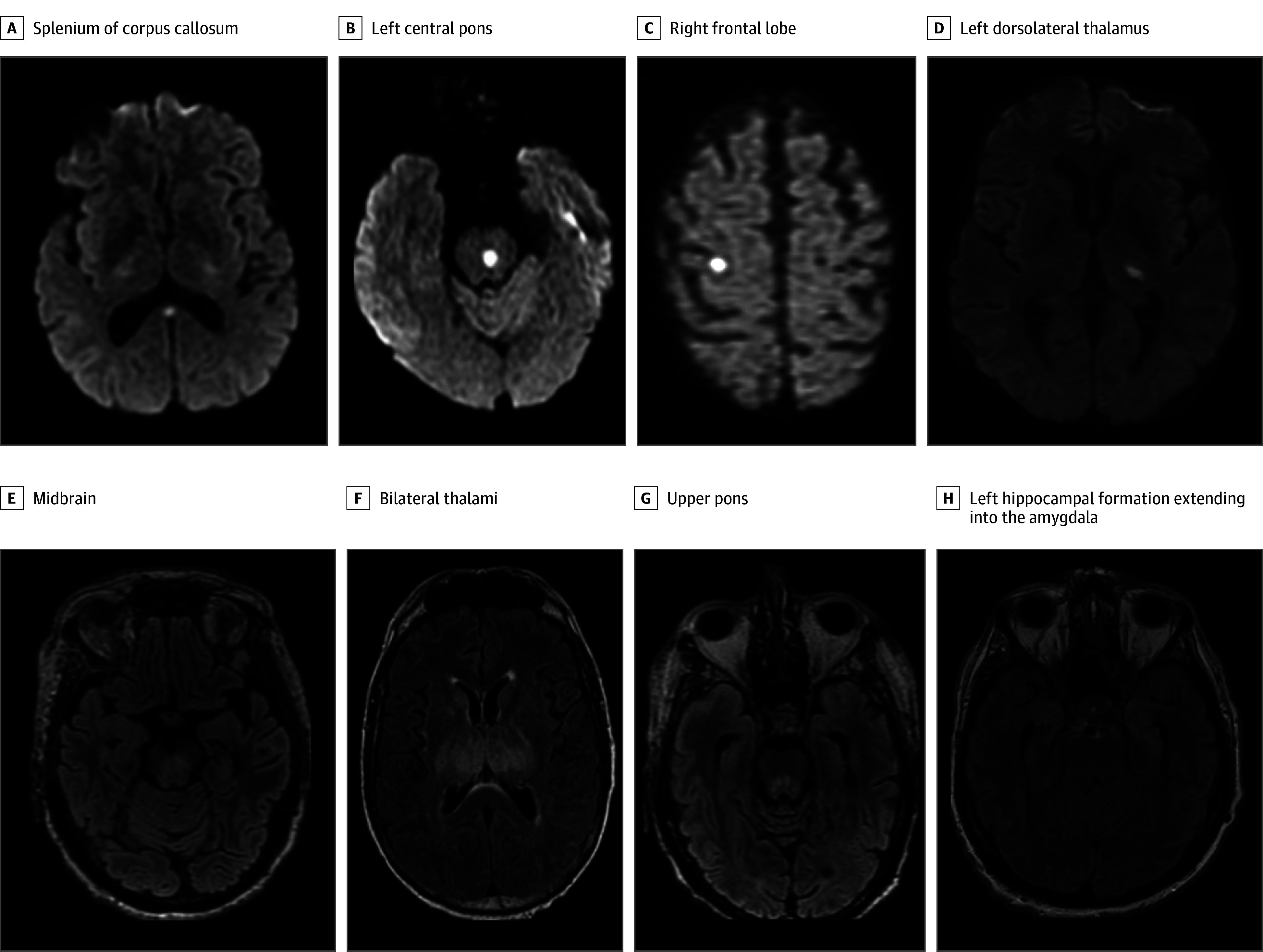
Diffusion-Weighted Imaging Brain Magnetic Resonance Imaging Sequences From Different Patients Showing Focal Areas of Diffusion Restriction A-D, Patients had an apparent diffusion coefficient correlate with decreased signal suggesting acute ischemia. E-G, Fluid-attenuated inversion recovery images of various patients showing areas of subtle increased fluid attenuated inversion recovery signal.

### Treatment Variables and Study Outcomes

Individuals with IS were significantly more likely to receive adjunctive therapies (IVIg and interferon alfa) (Table 2). Interferon alfa was administered to 16 individuals (37%) with IS vs 5 individuals (7%) without IS. Meanwhile, IVIg was administered to 24 individuals (56%) with IS vs 7 individuals (12%) without IS.

Hospital length of stay was significantly longer in individuals with IS than individuals without IS (median [IQR], 16 [11-26] days vs 8 [5-20] days). The crude 90-day mortality rate was 17 patients (15%) in the entire cohort and significantly higher in patients with IS (12 patients [28%]) than patients without IS (5 patients [7%]). Notably, the difference in mortality between groups persisted even after controlling for disease severity (GCS) in the multivariable regression model (adjusted hazard ratio [HR], 2.22; 95% CI, 1.07-4.27; *P* = .03) (Table 4).

**Table 4.** Regression Analysis for Associations With 90-Day Mortality in the Entire Cohort

Variable	Univariable analysis		Multivariable analysis	
	HR (95% CI)	*P* value	aHR (95% CI)	*P* value
Age, per 1-y increase	1.01 (0.99-1.05)	.25	NA	NA
Gender				
Male	1 [Reference]	.46	NA	NA
Female	0.75 (0.35-1.62)		NA	NA
Immunosuppression status				
Immunocompetent	1 [Reference]	.02	1 [Reference]	.03
Immunosuppressed	2.41 (1.19-4.88)		2.22 (1.07-4.63)	
Clinical seizure				
Absent	1 [Reference]	.71	NA	NA
Present	0.82 (0.29-2.34)		NA	NA
Fever at admission				
Absent	1 [Reference]	.60	NA	NA
Present	1.23 (0.57-2.65)		NA	NA
Headache				
Absent	1 [Reference]	.82	NA	NA
Present	0.92 (0.46-1.85)		NA	NA
Insomnia				
Absent	1 [Reference]	.004	NA	NA
Present	3.39 (1.47-7.86)		NA	NA
Myalgias				
Absent	1 [Reference]	.10	NA	NA
Present	0.49 (0.21-1.14)		NA	NA
Altered mental status				
Absent	1 [Reference]	.08	NA	NA
Present	2.05 (0.92-4.57)		NA	NA
Focal motor symptoms				
Absent	1 [Reference]	.52	NA	NA
Present	1.29 (0.60-2.78)		NA	NA
Myoclonus				
Absent	1 [Reference]	NA	NA	NA
Present	2.39 (0.92-6.20)	.08	NA	NA
Duration of symptoms, per 1-d increase	0.97 (0.91-1.03)	.33	NA	NA
ICU admission				
Absent	1 [Reference]	.001	NA	NA
Present	3.76 (1.67-8.37)		NA	NA
Coma, GCS<8				
Absent	1 [Reference]	<.001	1 [Reference]	.001
Present	3.79 (1.88-7.61)		3.63 (1.76-7.51)	
Mechanical ventilation				
Absent	1 [Reference]	<.001	NA	NA
Present	4.53 (2.04-10.09)		NA	NA
WNV specific treatment				
Not administered	1 [Reference]	.03	NA	NA
Administered	2.15 (1.07-4.34)		NA	NA
IVIg treatment				
Not administered	1 [Reference]	.002	NA	NA
Administered	3.03 (1.51-6.10)		NA	NA
Interferon treatment				
Not administered	1 [Reference]	.02	NA	NA
Administered	2.40 (1.16-4.97)		NA	NA
Positive Serum WNV IgM				
Absent	1 [Reference]	.045	1 [Reference]	.08
Present	0.42 (0.18-0.98)		0.47 (0.20-1.100)	
Positive CSF WNV IgM				
Absent	1 [Reference]	NA	NA	NA
Present	0.74 (0.22-2.50)	.62	NA	NA
CSF WNV PCR, positive	2.39 (0.92-6.22)	.07	NA	NA
Tracheostomy procedure				
Absent	1 [Reference]	NA	NA	NA
Present	2.51 (1.22-5.14)	.01	NA	NA
CSF glucose, per 1-mg/dL increase	1.01 (0.99-1.02)	.45	NA	NA
CSF protein, per 1-mg/dL increase	1.00 (1.00-1.01)	.36	NA	NA
CSF white blood cell count, per 1/μL increase	1.00 (1.00-1.00)	.79	NA	NA
Acute MRI abnormalities present				
Absent	1 [Reference]	.69	NA	NA
Present	1.23 (0.44-3.45)		NA	NA
WNV encephalomyelitis present				
Absent	1 [Reference]	.91	NA	NA
Present	1.25 (0.22-7.17)		NA	NA

Abbreviations: CSF, cerebrospinal fluid; GCS, Glasgow Coma Scale; ICU, intensive care unit; IgM, immunoglobulin M; IVIg, intravenous immunoglobulin; MRI, magnetic resonance imaging; NA, not applicable; PCR, polymerase chain reaction; WNV, West Nile virus.

IVIg administration was associated with an increased unadjusted hazard of mortality in the entire cohort (HR, 3.03; 95% CI, 1.51-6.10; *P* = .002), while this association was not seen when regression analysis was only performed in the subgroup of individuals with IS (HR, 1.24; 95% CI, 0.50-3.09; *P* = .64) (eTable in Supplement 1).

## Discussion

In this cohort study, we evaluated the clinical, laboratory, and neuroimaging characteristics, as well as outcomes in individuals with or without IS, among patients with NWNV infection. Consistent with prior findings, all patients with NWNV infection in our cohort presented in the summer and fall months.^11^ Interestingly, there was a 3-day delay from symptom onset to hospital presentation in individuals with IS compared with those without IS. While the exact cause of this delay is unclear, we speculate that a less robust immune response in these individuals may have masked the over clinical manifestations. Additionally, individuals without IS were more likely to present with pain-related symptoms, such as headache and myalgia, which could lead to earlier presentation, as opposed to the more subtle symptoms of myoclonus and confusion that were more common in individuals with IS.

Laboratory findings were similar between groups, with a few notable exceptions. CSF white blood cell count was significantly higher in patients without IS, while CSF WNV PCR testing was significantly more likely to have positive results in individuals with IS. These findings might indicate a more vigorous intrathecal immune response in patients without IS, effectively clearing the virus from the CSF. In contrast, patients with IS may experience an extended period of viral presence in the central nervous system.^12^

Brain MRI revealed T2 FLAIR hyperintensities in half of individuals with available imaging data, mostly involving the midbrain, pons, deep gray nuclei, and mesial temporal lobe. These findings are consistent with prior reports, including a case series of 16 patients in whom 50% were found to have abnormal MRI findings in the deep gray matter and or brainstem areas.^13^ In addition, autopsy-based studies have similarly demonstrated a predilection for the brain stem and deep gray nuclei in patients with NWNV infections.^14^ The exact reason for the affinity of WNV to these areas is unknown. Remarkably, abnormal T2 FLAIR changes were more common in individuals with IS than those without IS. These findings, while nonspecific,^13,15^ should raise suspicion for NWNV infection in patients presenting with aseptic meningitis or encephalitis in the summer or fall months.

While there are no currently approved treatments for NWNV disease beyond supportive care, adjunctive therapies (eg, IVIg, interferon alfa) were received by 42% of patients in the study cohort. Current Centers for Disease Control and Prevention guidelines do not support the use of these agents in NWNV infection.^16^ However, continued use of IVIg and interferon alfa is supported by a number of small studies and case reports demonstrating that it is safe and potentially beneficial, especially in individuals with IS.^9,17,18,19,20,21^ In our cohort, patients with IS were more likely to receive these therapies, possibly due to higher disease severity, prompting clinicians to consider some of these off-label therapeutic options in the absence of convincing data. Notably, we did find that IVIg use was associated with higher hazards of mortality in our cohort, which reflects bias of preferentially offering treatment to patients who are the most severely ill. This possibility is supported by our subgroup analysis of patients with IS only, in whom we found no association between IVIg use and all-cause mortality. In the absence of proven and effective therapies for NWNV infection, transient cessation or deescalation of immune-suppressive therapies is paramount to aid in immune reconstitution and improved outcomes.

Individuals with IS had worse outcomes compared with individuals without IS in this study. These findings are consistent with those of a prior study from Mayo Clinic Arizona, which similarly found a high mortality rate in individuals who underwent SOT.^9^ We hypothesize that the IS state itself confers increased risk of severe disease, which can partly explain this difference in outcomes. Other potential contributing factors could include delayed hospital presentation and higher rates of CSF viremia in patients with IS. Improved awareness and consideration for the possibility of atypical NWNV presentations among individuals with IS in high-risk areas could result in earlier hospitalizations and potentially improved outcomes.

This study has several strengths, including having the largest number of patients with NWNV infection reviewed thus far, to our knowledge. This study also includes patients from the large WNV outbreak of more than 1400 reported cases in Maricopa County, Arizona, in 2021, thus offering a unique opportunity to provide key insights of disease features and outcomes. Notably, in our anecdotal experience, this most recent WNV outbreak was different from previous years in a few key metrics, including onset of NWNV cases earlier in the year and persisting throughout the late fall months, as well as a higher rate of patients presenting with severe brainstem syndromes.

### Limitations

This study has several limitations that are worth noting. First, our search criteria, although robust, might have missed some patients with NWNV infection. We used *ICD-9* and *ICD-10* codes to identify patients, which captured most of the cases. However, patients with inappropriately coded disease at the time of discharge might have been missed. We acknowledge that a more sensitive way of identifying patients would have been to use microbiological and serology laboratory data to identify all patients with a positive WNV test result in serum or CSF, and then review medical records for inclusion. Furthermore, if a specific diagnosis related to WNV, such as myelitis or radiculitis, was not accurately documented or diagnosed in the patient’s health record, it would have precluded our ability to record such a diagnosis. Second, this is a medical record review study in which patients were classified retrospectively and therefore misclassification bias cannot entirely be excluded. However, this is mitigated by the fact that we collected all data from a robust electronic medical records system at a tertiary academic center where information is recorded in a consistent manner and laboratory tests for all patients were completed at a Mayo Clinic laboratory. The diagnoses were recorded as written in the patient records. Third, our dataset was subjected to multiple statistical testing for the multiple study outcomes, which amplifies the risk of false-positive findings. Fourth, referral bias is possible, with only the patients who were most severely ill referred to a tertiary facility such as ours, which can skew outcomes. Fifth, our dataset was insufficient to systematically assess mid-term to long-term functional disability outcomes of survivors, which would have provided a more complete picture of disease outcomes.

## Conclusions

In this cohort study of patients with NWNV infection, we found that individuals with NWNV infection commonly presented in the summer or fall, with clinical and laboratory features consistent with aseptic meningoencephalitis and MRI T2 FLAIR abnormalities commonly localized in the brain stem or deep gray areas. Individuals with IS were at an increased risk for poor outcomes, emphasizing the need for prompt initiation of supportive therapy. Although IVIg and interferon alfa therapy were associated with worse outcomes in our cohort, this observation warrants cautious interpretation due to potential treatment bias toward patients who were more severely ill. Our findings highlight the importance of raising awareness about NWNV disease among health care practitioners and individuals with IS. Furthermore, the development of effective antiviral therapies is crucial for improving outcomes in the high-risk subgroup of individuals with IS.

## Notes

Supplement 1. **eTable.** Regression Analysis for Factors Associated With 90-Day Mortality Among Patients With Immunosuppression **eFigure 1.** Flow Chart Showing Number of Participants Screened and Enrolled **eFigure 2.** Total Number of Neuroinvasive West Nile Virus Cases Admitted Across all the 3 Mayo Clinic Hospitals in Arizona, Florida, and Rochester Between July 2006 to September 2019, Grouped by the Months and Season of Presentation Independent of the Year

Supplement 2.
Data Sharing Statement
