# Supplementary material for: Neuroinvasive West Nile Virus Infection in Immunosuppressed and Immunocompetent Adults
Source: JAMA Netw Open. 2024 Mar 28;7(3):e244294. doi: 10.1001/jamanetworkopen.2024.4294 (PMC10979308; doi:10.1001/jamanetworkopen.2024.4294)
Supplement: Supplement 1. — eTable. Regression Analysis for Factors Associated With 90-Day Mortality Among Patients With Immunosuppression eFigure 1. Flow Chart Showing Number of Participants Screened and Enrolled eFigure 2. Total Number of Neuroinvasive West Nile Virus Cases Admitted Across all the 3 Mayo Clinic Hospitals in Arizona, Florida, and Rochester Between July 2006 to September 2019, Grouped by the Months and Season of Presentation Independent of the Year [file jamanetwopen-e244294-s001.pdf]

Supplemental Online Content

Mbonde AA, Gritsch D, Harahsheh EY, et al. Neuroinvasive West Nile virus infection in immunosuppressed and immunocompetent adults. *JAMA Netw Open*. 2024;7(4):e244294. doi:10.1001/jamanetworkopen.2024.4294

eTable. Regression Analysis for Factors Associated With 90-Day Mortality Among Patients With Immunosuppression
eFigure 1. Flow Chart Showing Number of Participants Screened and Enrolled
eFigure 2. Total Number of Neuroinvasive West Nile Virus Cases Admitted Across all the 3 Mayo Clinic Hospitals in Arizona, Florida, and Rochester Between July 2006 to September 2019, Grouped by the Months and Season of Presentation Independent of the Year

This supplemental material has been provided by the authors to give readers additional information about their work.

© 2024 Mbonde AA et al. *JAMA Network Open.*

Supplementary Table 1. Regression analysis for factors associated with 90-day mortality amongst those with immunosuppression

	Univariable analysis	
**Variable**	**HR (95% CI)**	**P-value**
Age, years, per unit increase	1.02 (0.98, 1.05)	0.321
Gender		
Male	REF	
Female	0.63 (0.24, 1.65)	0.344
Clinical Seizure		
Absent	REF	
Present	0.74 (0.22, 2.55)	0.638
Fever at admission		
Absent	REF	
Present	1.08 (0.41, 2.86)	0.872
Headache		
Absent	REF	
Present	0.92 (0.37, 2.27)	0.852
Insomnia		
Absent	REF	
Present	3.99 (1.30, 12.26)	0.016
Myalgias		
Absent	REF	
Present	1.23 (0.41, 3.73)	0.712
Altered mental status		
Absent	REF	
Present	1.80 (0.60, 5.47)	0.297
Focal motor symptoms		
Absent	REF	
Present	1.60 (0.63, 4.08)	0.324
Myoclonus		
Absent	REF	

© 2024 Mbonde AA et al. *JAMA Network Open.*

Present	1.52 (0.50, 4.59)	0.459
Duration of symptoms,(days), per unit increase	0.96 (0.89, 1.03)	0.270
ICU admission		
Absent	REF	
Present	3.89 (1.07, 12.66)	0.038
Coma, GCS < 8		
Absent	REF	
Present	3.47 (1.38, 8.72)	0.008
Mechanical ventilation		
Absent	REF	
Present	4.97 (1.44, 17.08)	0.011
WNV Specific Treatment		
Not administered	REF	
Administered	1.02 (0.39, 2.69)	0.969
IVIG treatment		
Not administered	REF	
Administered	1.56 (0.59, 4.12)	0.367
Interferon treatment		
Not administered	REF	
Administered	1.24 (0.50, 3.09)	0.641
Positive Serum WNV IgM		
Absent	REF	
Present	0.37 (0.14, 1.00)	0.050
Positive CSF WNV IgM		
Absent	REF	
Present	0.61 (0.24, 1.55)	0.295
CSF WNV PCR, positive	1.38 (0.41, 4.59)	0.601
Tracheostomy procedure done		
Absent	REF	
Present	1.99 (0.76, 5.16)	0.159

© 2024 Mbonde AA et al. *JAMA Network Open.*

CSF glucose, level mg/dl, per unit increase	1.01 (0.98, 1.03)	0.563
CSF protein, level mg/dl, per unit increase	1.01 (1.00, 1.012)	0.215
CSF white blood cell count, cell/mm3, per unit incre	1.00 (1.00, 1.01)	0.376
Acute MRI abnormalities present		
Absent	REF	
Present	0.48 (0.14, 1.59)	0.227
WNV encephalomyelitis present		
Absent	REF	
Present	0.98 (0.28, 3.40)	0.979

Abbreviations: ICU: Intensive Care Unit; GCS: Glascow Coma Scale, WNV: West Nile Virus; IVIG: Intravenous Immunoglobulin; CSF: Cerebrospinal Fluid; IgM: Immunoglobulin M; IgG: Immunoglobulin G; PCR: Polymerase chain reaction; MRI: Magnetic Resonance Imaging; EEG: Electroencephalogram

© 2024 Mbonde AA et al. *JAMA Network Open.*

eFigure 1: Flow chart showing number of participants screened and enrolled.

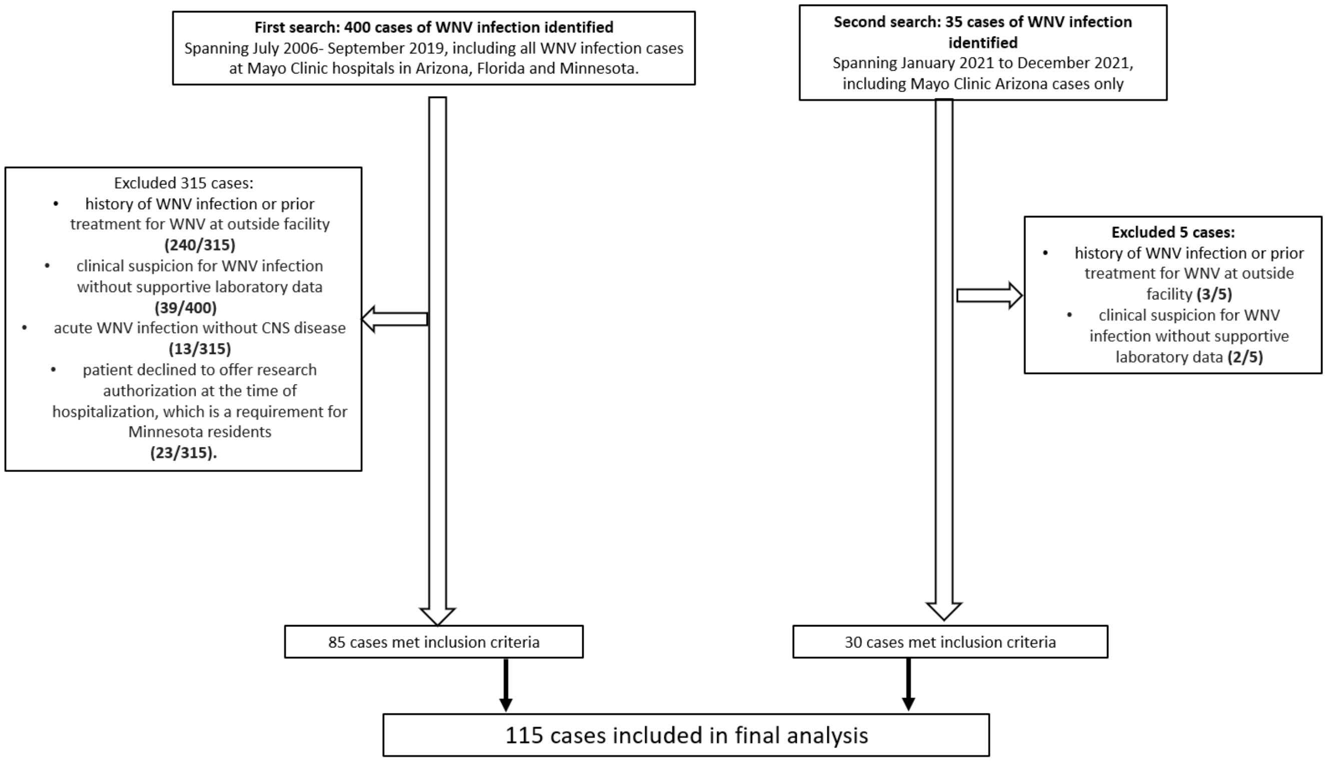

© 2024 Mbonde AA et al. *JAMA Network Open.*

eFigure 2: Total number of neuroinvasive west Nile virus cases admitted between July 2006 to September 2019, grouped by the months and season of presentation independent of the year.

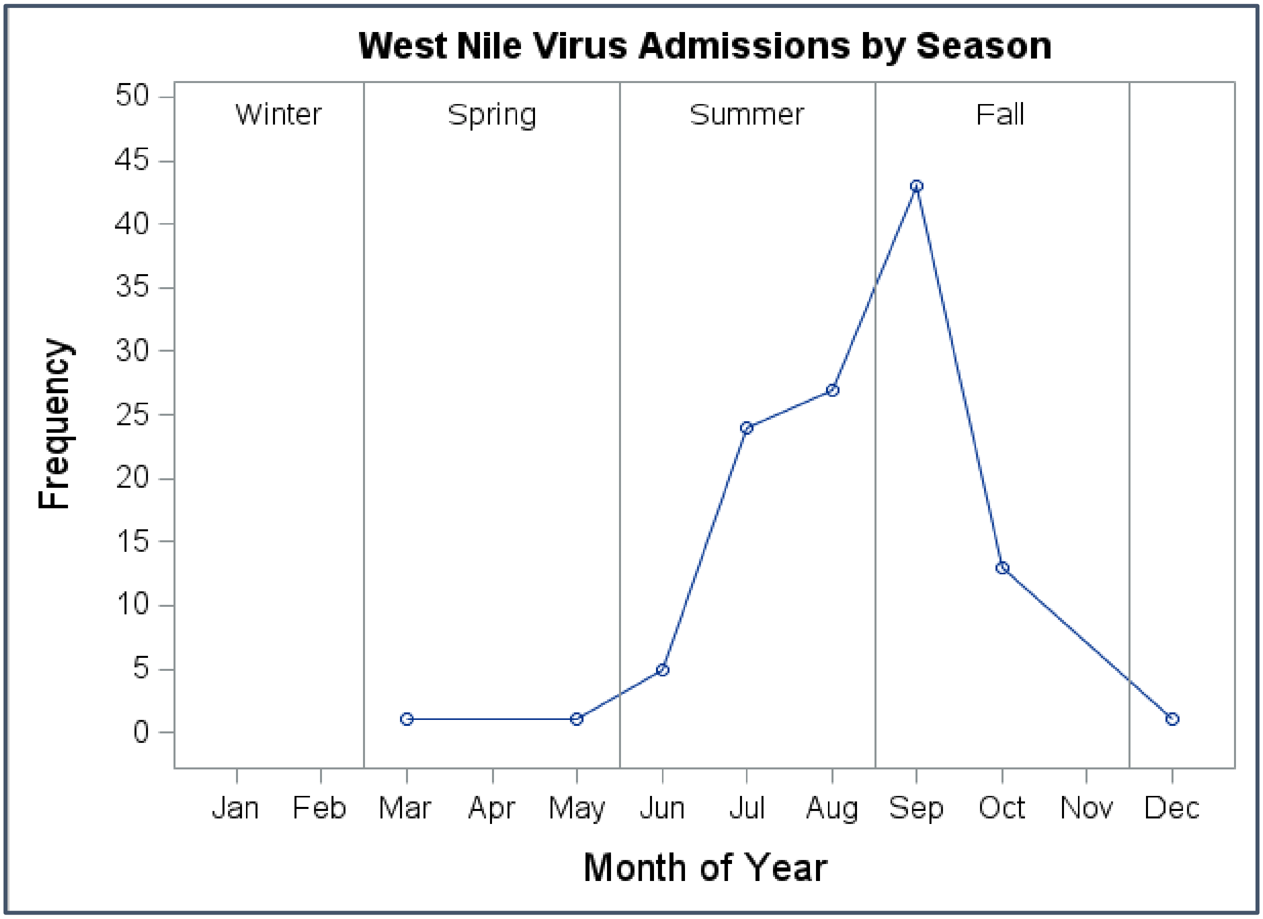

© 2024 Mbonde AA et al. *JAMA Network Open.*
