# Supplementary material for: Neuroinvasive West Nile Virus Infection in Immunosuppressed and Immunocompetent Adults
Source: JAMA Netw Open. 2024 Mar 28;7(3):e244294. doi: 10.1001/jamanetworkopen.2024.4294 (PMC10979308; doi:10.1001/jamanetworkopen.2024.4294)
Supplement: Supplement 2. — Data Sharing Statement [file jamanetwopen-e244294-s002.pdf]

Data Sharing Statement

Mbonde. Neuroinvasive West Nile Virus Infection in Immunosuppressed and Immunocompetent Adults. *JAMA Netw Open*. Published April 01, 2024. doi:10.1001/jamanetworkopen.2024.4294

Data
Data available: Yes
Data types: Deidentified participant data
How to access data: Please send requests to mbonde.amir@mayo.edu and grill.marie@mayo.edu
When available: With publication

Supporting Documents
Document types: Statistical/analytic code
How to access documents: mbonde.amir@mayo.edu
When available: With publication

Additional Information
Who can access the data: Anyone with a reasonable request. Upon approval.
Types of analyses: For any purpose
Mechanisms of data availability: After approval of a request
Any additional restrictions: Mayo clinic approval will be required
